# The Protective Potential Role of ACE2 against COVID-19

**DOI:** 10.1155/2023/8451931

**Published:** 2023-05-26

**Authors:** Fereshteh Golab, Gelareh Vahabzadeh, Leila SadeghRoudbari, Arefeh Shirazi, Robabeh Shabani, Sara Tanbakooei, Lida Kooshesh

**Affiliations:** ^1^Cellular and Molecular Research Center, Iran University of Medical Sciences, Tehran, Iran; ^2^Razi Drug Research Center, Department of Pharmacology, School of Medicine, Iran University of Medical Sciences, Tehran, Iran; ^3^Department of Cellular and Molecular Biology, Islamic Azad University, Tehran North Branch, Tehran, Iran

## Abstract

Due to the coronavirus disease 2019 (COVID-19), researchers all over the world have tried to find an appropriate therapeutic approach for the disease. The angiotensin-converting enzyme 2 (ACE2) has been shown as a necessary receptor to cell fusion, which is involved in infection due to severe acute respiratory syndrome coronavirus 2 (SARS-CoV-2). It is commonly crucial for all organs and systems. When ACE2 is downregulated via the SARS-CoV-2 spike protein, it results in the angiotensin II (Ang II)/angiotensin type 1 receptor axis overactivation. Ang II has harmful effects, which can be evidenced by dysfunctions in many organs experienced by COVID-19 patients. ACE2 is the SARS-CoV-2 receptor and has an extensive distribution; thus, some COVID-19 cases experience several symptoms and complications. We suggest strategy for the potential protective effect of ACE2 to the viral infection. The current review will provide data to develop new approaches for preventing and controlling the COVID-19 outbreak.

## 1. Introduction and Background

In December 2019, the severe acute respiratory syndrome coronavirus 2 (SARS-CoV-2) resulted in coronavirus disease 2019 (COVID-19), first identified in Wuhan, China, with a rapid spread in China and 27 other countries with high mortality. There is no definite treatment for this disease, and those available are restrictions on travelling, isolating the patients, and supportive healthcare; however, several medications have been examined [1–3]. Identification of the underlying pathobiology of the disease is helpful. In the current study, the angiotensin-converting enzyme 2 (ACE2) receptor, a special target of SARS-CoV-2, will be considered and reviewed.

## 2. Angiotensin-Converting Enzyme (ACE) and Its Homolog (ACE2)

The renin-angiotensin system (RAS) regulates the cardiovascular and kidney functions and contributes to the pathophysiology of cardiovascular and kidney disorders [4, 5]. The RAS effector peptides can be generated and degraded via several enzymatic reactions that indicate their concentration in the plasma and many tissues [6]. Angiotensin (Ang) processing begins by angiotensinogen hydrolysis through protease renin for generating Ang I (Ang1–10) [7]. ACE due to its peptidase-dependent activity can convert Ang I to octapeptide Ang II (Ang1–8) [8].

Ang1–8 is the main RAS mediator and is associated with many physiological events. It enhances vascular smooth muscle contraction, which increases the systemic vascular resistance. It is also able to initiate sodium reabsorption within the kidneys through the stimulation of aldosterone release and acting as the main mediator of the kidney tubuloglomerular feedback mechanism [9]. Moreover, it has strong proinflammatory and proangiogenic effects [10, 11]. It attaches to AT-1 and AT-2 receptors; the former mediates its vasoconstrictive, proliferative, and proinflammatory effects [8].

**Figure 1:**
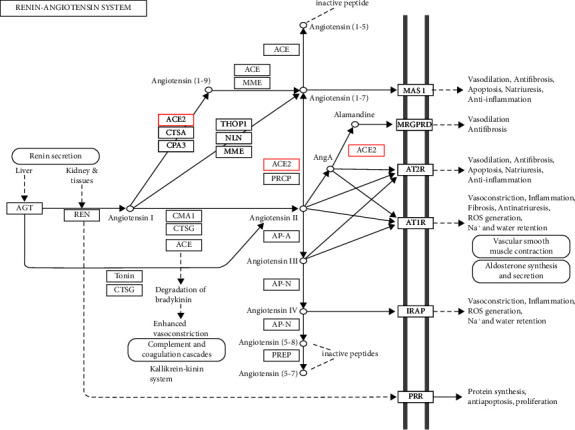
Detailed representation of the renin-angiotensin system cascade. ACE, angiotensin-converting enzyme; Ang, angiotensin; AT 1, Ang II type 1 receptor; AT2, Ang II type 2 receptor; Mas, Ang-(1–7) receptor.

In recent years, ACE2 as a homolog of ACE was detected [12–14]. It mainly acts as a mono-carboxypeptidase that preferably hydrolyzes between proline and a hydrophobic/basic C-terminal amino acid. An enzymatic reaction, which is successfully catalyzed via ACE2, involves Ang1–8 degradation through the removal of its *C*-terminal phenylalanine for generating Ang1–7 [15–17] that is characterized by vasodilatory, antiproliferative, antiangiogenic, and anti-inflammatory properties [18]. G protein-coupled Mas receptors mediate its effects [19]. Also, Ang1–7 suppresses the activities of the carboxy-terminal domain of ACE, by which it prevents ACE against fully acting on Ang I and bradykinin [6, 20] (Figure 1).

Considering the mentioned mechanisms, the simultaneous reduction in Ang1–8 and an elevation of Ang1–7 positively affect many diseases [6]. Since these beneficial effects can be achieved by ACE2, the enhanced function of this enzyme may provide effective strategies to treat illnesses with pathologically elevated Ang1–8 and/or reduced Ang1–7 because of an imbalanced RAS.

Regarding the negative regulation of RAS by ACE2, this enzyme is currently a functional receptor for SARS-CoV-2 [21–23]. Coronaviruses can efficiently recognize ACE2, and the SARS-CoV-2 spike protein possesses a high binding affinity for human ACE2 [24, 25]. These viruses use ACE2 because of its cellular entry to the host cell as well as for the downregulation of ACE2 expression [26] that is involved in the acute respiratory distress syndrome (ARDS) and severe acute respiratory syndrome (SARS) pathogenesis [27].

The epidemiological findings suggest an increase in ACE2 expression in youths than aged individuals [28]. The reduced ACE2 amount in aged cases possibly is associated with the prevalence of consequences in aging [21]. Bioinformatic analysis data of the genomics and transcriptomics gene expression in human [29] demonstrated that expression of ACE2 decreases with ageing in many tissues. According to Chen et al., ACE2 expression decreases with age in various organs including blood, adrenal gland, colon, nervous system, adipose tissues, and salivary gland [29]. Several studies have demonstrated that ageing is linked with reduced expression of ACE2 in both experimental and human models [29–31]. Xudong et al. [31] investigated the impact of ageing on ACE2 expression in lung epithelial cells and found that the older group exhibited significantly lower levels of ACE2 expression compared to the young group [31]. Moreover, ACE2 restricts the macrophage expression in many proinflammatory cytokines *in vitro*, such as tumor necrosis factor-*α* (TNF-*α*) and interleukin-6 (IL-6) [32]. However, regarding COVID-19, ACE2 is downregulated by the virus, which enhances the macrophage expression [33], evidenced by macrophage activation. ACE2 has a high level of expression in the luminal area of tubular epithelial cells in the kidneys [34], as well as cardiomyocytes in the heart [35]. It is also detectable in the gut and the lungs [36]. Dysfunctions of different organs in COVID-19 cases are explainable by the elevated level of ACE2 expression. Therefore, SARS-CoV-2 infection downregulates ACE2 and causes the Ang II involvement [21]. It is assumed that ACE2 has a protective function in different organs, and ACE2 downregulation in SARS-CoV-2 infection has deleterious effects [37]. The overactivation of the angiotensin (Ang) II/AT1R axis caused by the SARS-CoV-2 spike protein's downregulation of ACE2 may be the reason for the harmful effects of Ang II, which could potentially clarify the multiorgan dysfunction observed in patients. [37]. Due to its extensive distribution throughout various organs, ACE2 has the ability to counteract the activation of the conventional RAS system, thus safeguarding against hypertension, diabetes, cardiovascular disease, and organ damage [38]. Accordingly, we can propose potential treatments to obtain better outcomes in severe COVID-19 patients.

## 3. Role of ACE2 in Lung Protection

In the respiratory tract, ACE2 expression mainly occursin the alveolar/bronchiolar epithelium, endothelium, and smooth muscle cells of lung vessels [39]. Cell differentiation as well as ACE2 expression levels determine the vulnerability of human airway epithelial cells to infections [40]. In the respiratory system, treatment with recombinant ACE2 was effective for lung diseases and survival of patients with virus-induced ARDS and SARS [6, 41, 42]. SARS-CoV-2 infection is associated with the ACE2 depletion from the cell surface and the loss of ACE2-mediated tissue protection [21].

The therapeutic effects of recombinant human ACE2 (rhACE2) have been recently investigated in different acute and chronic animal diseases linked to enhanced Ang1–8 concentrations or dysregulated RAS. In ACE2-knockout mice receiving Ang1–8, rhACE2 prevented Ang1–8-related arterial hypertension, oxidative stress, and tubulointerstitial fibrosis [43]. It also inhibited pathological hypertrophy, myocardial fibrosis, and diastolic dysfunction [44], while reducing the diabetic nephropathy progression [45]. Also, rhACE2 suppressed the liver fibrosis development in bile duct ligation and chemically-induced liver fibrosis in a mouse model [46]. Moreover, rhACE2 systemic administration caused an improvement in the pulmonary blood flow as well as blood oxygenation in a lipopolysaccharide (LPS)-associated ARDS model in piglets [47].

According to Kuba et al. [48], SARS-CoV-2 downregulated ACE2 protein (but not ACE) in mice through attachment to its spike protein, leading to severe lung injury. Therefore, excessive amounts of ACE2 in a competitive manner are attached to SARS-CoV-2 for neutralizing the virus as well as rescuing cellular ACE2 activity that has a negative regulatory role for RAS to protect the lungs against injury [49, 50]. It is known that the increased ACE activity and reduced ACE2 accessibility cause lung injury in acid- and ventilator-related lung injury [49, 51, 52]. Therefore, administrating the soluble type of ACE2 has a dual function: (1) slow entrance of the virus into cells, leading to viral spread [53, 54]; and (2) protection of the lungs against damage [48].

**Figure 2:**
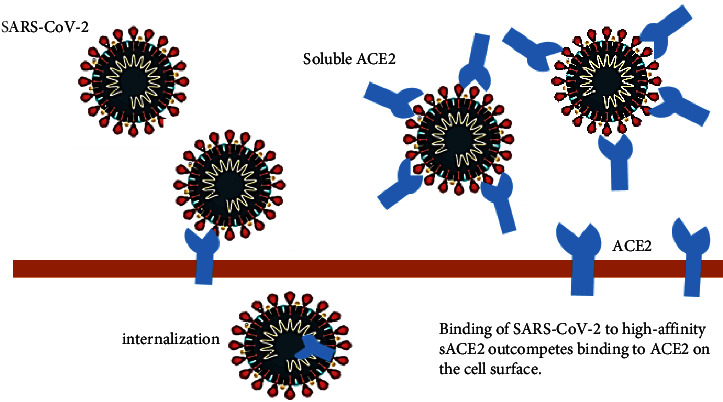
Binding of SARS-CoV-2 to high-affinity sACE2 outcompetes binding to ACE2 on the cell surface.

It has been shown that the rhACE2 protein (APN01 and GSK2586881) has no harmful hemodynamic impacts in normal cases and some cases with ARDS [6, 42, 55]. In this regard, Haschke et al. carried out the first single-dose escalation and tolerability research on human to assess the pharmacokinetics and pharmacodynamics of rhACE2 following its intravenous admonition in normal humans [6]. Moreover, Khan et al. showed that infusion of GSK2586881, a recombinant form of rhACE2, resulted in expected changes of RAS biomarkers and was well-tolerated by ARDS patients [42]. Also, Monteil et al. demonstrated that human recombinant soluble ACE2 (hrsACE2) remarkably blocked the early stages of SARS-CoV-2 infection [56]. Alhenc-Gelas and Drueke showed that ARDS patients well-tolerated sACE2 in clinical phase I and II trials. They suggested that sACE2 has additional effectiveness on the lungs and different organs, such as the kidneys [57]. The development of human recombinant soluble ACE2, also known as hrsACE2, aims to reduce lung injury and prevent multiple organ dysfunction. This is achieved by competing with membrane-bound ACE2, thereby reducing the SARS-CoV-2 entrance into target cells [58] (Figure 2). In addition, the growth of SARS-CoV-2 virus is significantly decreased by approximately 1000–5000 times in cell culture, engineered human blood vessels, and kidney organoids upon administration of the engineered human recombinant soluble ACE2 (hrsACE2) [56].

## 4. Conclusion

The exciting potential of hrsACE2 as a protective measure against viral infections, including COVID-19, has been revealed in recent research. While these findings hold great promise, further investigation is crucial to fully explore the therapeutic possibilities of this innovative approach.

## Data Availability

The data used to support the findings of this are available on reasonable request from the corresponding author.
